# Ultra-High-Throughput Screening of an *In Vitro*-Synthesized Horseradish Peroxidase Displayed on Microbeads Using Cell Sorter

**DOI:** 10.1371/journal.pone.0127479

**Published:** 2015-05-20

**Authors:** Bo Zhu, Takuro Mizoguchi, Takaaki Kojima, Hideo Nakano

**Affiliations:** Laboratory of Molecular Biotechnology, Graduate School of Bioagricultural Sciences, Nagoya University, Furo-cho, Chikusa-ku, Nagoya, Japan; Vrije Universiteit Brussel, BELGIUM

## Abstract

The C1a isoenzyme of horseradish peroxidase (HRP) is an industrially important heme-containing enzyme that utilizes hydrogen peroxide to oxidize a wide variety of inorganic and organic compounds for practical applications, including synthesis of fine chemicals, medical diagnostics, and bioremediation. To develop a ultra-high-throughput screening system for HRP, we successfully produced active HRP in an *Escherichia coli* cell-free protein synthesis system, by adding disulfide bond isomerase DsbC and optimizing the concentrations of hemin and calcium ions and the temperature. The biosynthesized HRP was fused with a single-chain Cro (scCro) DNA-binding tag at its N-terminal and C-terminal sites. The addition of the scCro-tag at both ends increased the solubility of the protein. Next, HRP and its fusion proteins were successfully synthesized in a water droplet emulsion by using hexadecane as the oil phase and SunSoft No. 818SK as the surfactant. HRP fusion proteins were displayed on microbeads attached with double-stranded DNA (containing the scCro binding sequence) via scCro-DNA interactions. The activities of the immobilized HRP fusion proteins were detected with a tyramide-based fluorogenic assay using flow cytometry. Moreover, a model microbead library containing wild type *hrp* (WT) and inactive mutant (MUT) genes was screened using fluorescence-activated cell-sorting, thus efficiently enriching the WT gene from the 1:100 (WT:MUT) library. The technique described here could serve as a novel platform for the ultra-high-throughput discovery of more useful HRP mutants and other heme-containing peroxidases.

## Introduction

The C1a isoenzyme of horseradish peroxidase (HRP) is the most abundant isoenzyme derived from horseradish (*Armoracia rusticana*). It is an important heme-containing enzyme that can utilize hydrogen peroxide (H_2_O_2_) to oxidize a wide variety of inorganic and organic compounds. This enzyme has been widely used in biochemical assays for the detection of H_2_O_2_ [1] and other target molecules after being coupled with the appropriate H_2_O_2_-generating enzymes (e.g., glucose oxidase [2] and monoamine oxidase [3]). HRP conjugated with antibodies or avidin derivatives is commonly used in detection techniques, such as western blotting [4], enzyme-linked immunosorbent assays [5], and immunohistochemical assays [6].

The amino acid sequence of mature HRP was first obtained by Welinder et al. in 1976 [7]. In 1988, Fujiyama et al. reported three cDNA sequences of HRP [8], and one of them contained a gene encoding the enzyme harboring the same amino acid sequence reported by Welinder. The S1 File shows the amino acid sequence (308 residues) used in our study of *in vitro* synthesis of mature HRP. From 1989 to 1999, several groups used the cDNA gene or two other synthetic genes to express HRP in *E*. *coli*. However, the HRP obtained in these ways always accumulated in inclusion bodies [9–15].

Site-directed mutagenesis and directed evolution techniques were used to engineer HRP suitable for various practical applications, including fine chemicals synthesis, medical diagnostics, and bioremediation [16]. Using directed evolution, researchers tried to generate HRP mutants that were suitable for biotechnological applications, by targeting properties such as activity [17,18], thermal stability [19], and resistance to H_2_O_2_ inactivation [19].

In order to perform the *in vivo* directed evolution of enzymes efficiently, proteins should be produced as active forms in the host cells. Morawski et al. used a *Saccharomyces cerevisiae* expression system for directed evolution of HRP. Interestingly, the generated mutant showed a 5.4-fold higher specific activity toward ABTS [2,2′-azinobis(3-ethylbenzthiazoline-6-sulfonic acid)] than did the wild type [17]. Agresti et al. used yeast cell-surface display coupled with a microfluidic-emulsion screening system, which increased the catalytic rate of the HRP mutants 10-fold relative to the wild type by introducing random mutations and screening with fluorescence-activated cell-sorting (FACS) [18]. In these studies, the host chosen for HRP expression was yeast instead of *E*. *coli*, because, in yeast, soluble active enzyme can be produced without refolding from inclusion bodies. However, the expression levels in yeast are relatively low (approximately 600 μg/L in ref. [17]) and the secreted enzyme undergoes post-translational hyperglycosylation. In addition, the size of the mutant library used for a high-throughput screening (HTS) method based on an *in vivo* expression system is innately limited by the efficiency of the transformation procedure.

The above problem could be solved by employing cell-free protein synthesis (CFPS) for HTS. CFPS, also known as *in vitro* protein synthesis or *in vitro* transcription/translation, was first developed in the 1960s [20]. This system uses cell extracts containing ribosomes and other factors, such as translation factors, tRNA, co-factors, amino acids, energy and etc., for transcription and translation, and rapidly synthesizes individual proteins from DNA/RNA templates in a tube. CFPS is advantageous compared to the *in vivo* expression of recombinant proteins. It can produce proteins within a few hours by using PCR products as templates. Moreover, cytotoxic proteins can also be generated in a cell-free system. Depending on the characteristics of different proteins, the synthesis conditions (including redox conditions, cofactors, and chaperons) can be controlled easily. Moreover, by engineering the energy generation pathway in a cell-free reaction mixture, the productivity of dual emission green fluorescent protein in an *E*. *coli*-based CFPS system can reach up to 2.3 mg/mL according to a recent report [21]. This yield is comparable to that obtained by *in vivo* expression.

A heme-containing enzyme, manganese peroxidase (MnP), was produced in an active form in our laboratory using an *E*. *coli* CFPS system. Its H_2_O_2_ stability was improved by screening a mutant library constructed using single-molecule-PCR-linked *in vitro* expression (SIMPLEX) [22]. Subsequently, by optimizing the reaction conditions and by adding disulfide bond isomerase, we successfully synthesized MnP with a higher specific activity than the commercial wild type enzyme, thereby suggesting that CFPS could be used as a preparative method for the efficient synthesis of disulfide bond-containing metalloenzymes, such as HRP [23].

CFPS is the basis of the bead display technology, which is an *in vitro* display technology linking the genotype and phenotype of a target protein on the same microbead by using emulsion PCR and emulsion CFPS [24,25]. The compartmentalization using emulsion droplets makes it possible to amplify a single DNA molecule on microbeads or to synthesize protein from template DNA on a single microbead, without contamination of each enzyme mutant from other microbeads. The bead display has been utilized in the development of *in vitro* ultra-high-throughput screening (uHTS) for the directed evolution of enzymes. Stapleton and Swartz developed a bead display-based *in vitro* uHTS method for [FeFe] hydrogenase [26]. They showed that microbeads displaying the *in vitro* synthesized active hydrogenase could be isolated using FACS and they used this method to enrich the positive microbeads from a 1:20 (positive control:negative control) model library.

**Fig 1:**
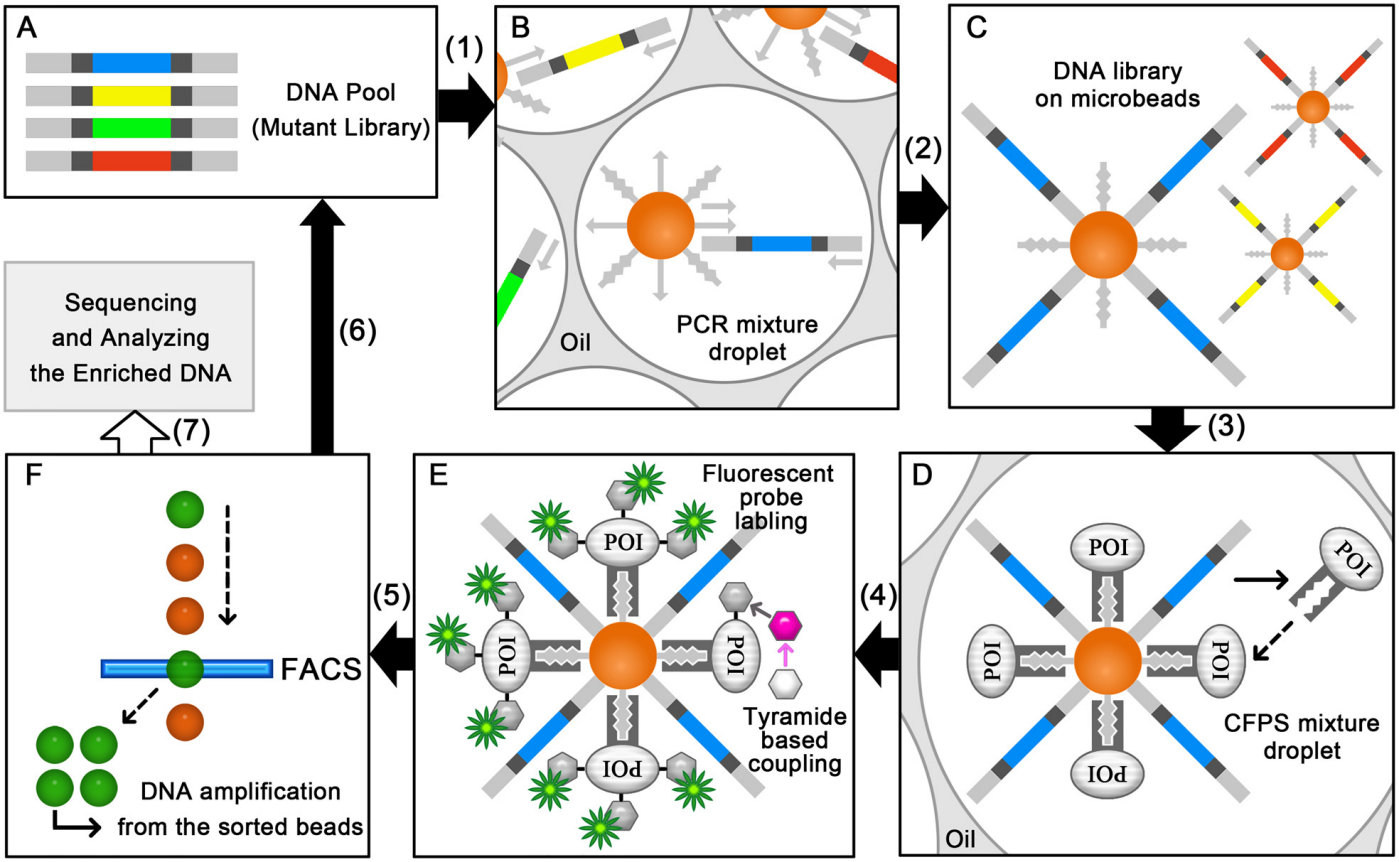
Scheme showing the principle of the bead display-based uHTS platform for peroxidase. Step 1: A DNA pool (panel A) is diluted with the PCR mixture containing the primer/scaffold hairpin-immobilized microbeads to less than one template DNA per droplet after emulsification. The template DNA is amplified on the microbead using emulsion PCR (panel B). Step 2: The DNA library on the microbeads (panel C) is recovered via emulsion disruption. Step 3: The recovered microbeads are diluted with the CFPS mixture to less than one microbead per droplet after emulsification. From the template DNA on the microbead, the peroxidase is synthesized by CFPS in emulsion and immobilized on the same microbead via the scCro-DNA interaction inside the droplets (panel D). In this way, the linkage of the genotype and phenotype on the same microbead is achieved. Step 4: The enzyme-DNA library on the microbeads is recovered from the emulsion, followed by a tyramide-based fluorogenic assay (panel E; POI: protein of interest). During this assay, the peroxidase catalyzes the conversion of the biotin-labeled tyramide to the short-lived tyramide radical that forms a covalent bond with a nearby tyrosine or tryptophan on the HRP surface. Then, streptavidin-Cy5 is used to obtain a fluorescent signal from the biotin. Step 5: After the fluorogenic assay, the microbeads are subjected to a selection process by using FACS (panel F). The microbeads with the peroxidase mutant of interest and the corresponding DNA are separated according to the relative strengths of their fluorescent intensities. Step 6: The template DNA on these selected microbeads are amplified to generate a new DNA pool with a higher ratio of the positive clone, and then subjected to another round of screening. Step 7: The sequences of the enriched DNA templates are analyzed after several rounds of screening.

In this study, we first established the *E*. *coli*-based CFPS, which is the prerequisite for the uHTS platform for HRP (Fig 1). Subsequently, HRP was synthesized as a fusion protein with an N-terminal and C-terminal single-chain Cro DNA-binding tag (scCro-tag) [27]. The tag present at both of these positions increased the solubility of HRP. Next, a tyramide-based fluorogenic assay system was developed for the *in vitro* screening system based on the bead display technique, in which the activity of the HRP immobilized on a magnetic microbead can be detected using flow cytometry. Finally, we validated this easy and high-throughput assay by screening a model microbead library bound to genes and proteins of wild type HRP (WT) and inactive mutant (MUT). An efficient enrichment of the wild type gene from a 1:100 library (WT:MUT) was achieved.

## Materials and Methods

### Optimization of the codon usage of the *hrp* gene for CFPS

The codon adaptation index (CAI) of the two synthetic genes and the cDNA gene mentioned above were calculated [28]. However, these CAIs were <0.65, indicating that the expression level might not be ideal. Therefore, a web server, OPTIMIZER [29], was used to optimize the codon usage of the DNA sequence and the GC contents of *hrp* to increase its expression level in *E*. *coli*, according to the CAI. The guided random method was used to generate the optimized sequence by choosing codons at random, based on the frequencies of use of each codon in the reference set. The generated sequence was recorded only when its CAI was >0.8 and when the GC content of all the regions was between 30% and 70%. Finally, four sequences were collected, and the free energies of their predicted mRNA secondary structures were calculated using a web server (http://www.genebee.msu.su/services/rna2_reduced.html). The candidate with the highest free energy was selected.

Then, a codon optimized 3×HA-tag and 8×Gly linker [30] were designed and fused to the N-terminus of HRP, and a 6×His-tag was added to the C-terminus of the enzyme. Finally, the CAI of the optimized DNA sequence of HRP with tags was equal to 0.8, and the GC content was equal to 56.48%. The sequence of the T7 promoter, the ribosome-binding site, and the T7 terminator from the pET-23b(+) vector (Merck Millipore, Billerica, MA, USA) were added to the HRP sequence. The entire optimized synthetic gene for the CFPS is shown in S2 File, and the S1 Fig shows the schematic diagram of the gene construction.

### Synthesis of templates for cell-free synthesis of HRP

The plasmid pUCIDT-KAN: T7P-3HA-G8-HRP-HIS-T7T containing the designed *hrp* gene was synthesized by Integrated DNA Technologies (Coralville, IA, USA). The DNA template, which contained the gene of the mature HRP with tags, T7 promoter, and T7 terminator, was amplified from the plasmid (1 ng) described above in a 20-μL PCR mixture with 0.05 U/μL *Pyrobest* DNA polymerase (Takara, Shiga, Japan) and 0.5 μM of each primer (Fw1: 5′-cgatc ccgcg aaatt aatac-3′ and R1: 5′-tccgg atata gttcc tcctt tcag-3′) using the following temperature sequence: preheating at 94°C for 3 min; followed by 25 cycles of 94°C for 15 s, 50°C for 15 s, and 72°C for 90 s, with an additional extension at 72°C for 7 min. The PCR products were purified with the QIAquick PCR Purification Kit (Qiagen, Hilden, Germany).

Via In-Fusion HD cloning (Takara), the scCro-tag was fused to HRP together with the T7-tag (N-terminal), GSGGGS linker (between HRP and scCro-tag), HA-tag (after linker), FLAG-tag (C-terminal) and 6×His-tag (after FLAG-tag). These epitope tags can be used for the purification of the HRP fusion and the detection of the HRP fusion displayed on microbeads. The pRSET B vector (Life Technologies, Carlsbad, CA, USA) was used to construct the plasmids (pRSET-HRP-scCro and pRSET-scCro-HRP) for CFPS of the HRP/scCro fusions. The sequence from T7 promoter to T7 terminator of the plasmid pRSET-HRP-scCro is shown in S3 File, and the S2 Fig shows the schematic diagram of the gene construction. The DNA templates T1 for CFPS of HRP/scCro fusion proteins were amplified from the plasmid (10 ng) mentioned above in a 20-μL PCR mixture with 0.05 U/μL *Pyrobest* DNA polymerase (Takara) and 0.5 μM of each primer (P2-F1-Fw1: 5′-ctgcc ccggg ttcct cattc tatct cgatc ccgcg aaatt aatac-3′ and P1-R1: 5′-ccact acgcc tccgc tttcc tctct atgtc cggat atagt tcctc ctttc ag-3′) using the following temperature sequence: preheating at 94°C for 1 min; followed by 20 cycles of 94°C for 15 s, 50°C for 30 s, and 72°C for 2 min, with an additional extension at 72°C for 7 min. The PCR products were purified with the QIAquick PCR Purification Kit (Qiagen).

### Cell-free protein synthesis of HRP

The *E*. *coli* extract is the most important part in a CFPS reaction mixture, because it contains the components required for the translation procedure, such as ribosomes, aminoacyl-tRNA synthetases, initiation, elongation and termination factors, etc. The S30 extract (*E*. *coli* lysate centrifuged at 30,000 × *g*) and the disulfide bond isomerase DsbC were prepared according to our previous report [23]. The cell-free protein synthesis mixture was prepared on ice containing 56.4 mM Tris-acetate (pH 7.4); 1.20 mM ATP; 1.13 mM each of GTP, UTP, and CTP; 60 mM sodium creatine phosphate; 0.75–2.34 mM of each of the 20 amino acids; 4% (w/v) polyethylene glycol 6000; 34.6 μg/mL folinic acid; 35.9 mM ammonium acetate; 0.17 mg/mL *E*. *coli* tRNA; 0.15 mg/mL creatine kinase; 10 μg/mL rifampicin; 10 mM magnesium acetate; 100 mM potassium acetate; 2 mM calcium acetate; 16.7 μM hemin; 1 mM GSH (reduced glutathione); 0.1 mM GSSG (oxidized glutathione); 200 μg/mL disulfide bond isomerase DsbC; 28.3% (v/v) *E*. *coli* S30 extract (strain BL21 Star DE3, Life Technologies, containing T7 RNA polymerase); and 6.67 ng/μL of the DNA template. The reaction was incubated at 21°C for 3 h. To visualize the synthesized protein, 0.15 μL of a fluorescently labeled lysine tRNA (FluoroTect GreenLys tRNA, Promega, Tokyo, Japan) was added to 30 μL of the CFPS reaction mixture. The soluble and insoluble HRP in 15 μL of the CFPS reaction mixture were analyzed with fluorographic SDS-PAGE. The fluorographic SDS-PAGE detection protocol has been described in our previous report [23]. The protein bands in the background might be the truncated fusion proteins by proteinases, nascent peptides or read-through proteins.

### Activity assay of HRP (OPD assay)

*o*-Phenylenediamine (OPD) was used as the substrate for the HRP activity assay. The diluted enzyme (1 μL) was mixed with 99 μL of the assay mixture containing 2 mg/mL OPD and 200 μM H_2_O_2_, and incubated at room temperature for 10 min. After incubation, 50 μL of 1 M sulfuric acid was added to stop the reaction. The absorption at 495 nm was read using a microplate reader (SpectraMax 250, Molecular Devices, CA, USA).

### *In situ* activity assay of HRP synthesized in the emulsion droplets

During this assay, 20 μL of the CFPS mixture was added to 400 μL of hexadecane with 3% w/v SunSoft No. 818SK (Taiyo Kagaku, Yokkaichi, Japan) and emulsified by vortexing for 1 min at room temperature, followed by a 3-h incubation at 21°C. The substrate delivery method was established based on the published ion delivery method [31]. During the procedure, 2 μL of the substrate delivery mixture containing 1 mM Amplex Red (Life Technologies), 900 μM H_2_O_2_, and 10% dimethyl sulfoxide, was added to 420 μL of the emulsion containing CFPS in the aqueous phase. The delivery was achieved by vortexing for 5 s and incubating for 10 min at room temperature. After the incubation, the emulsion droplets were observed directly using a confocal laser scanning microscope (LSM 5 PASCAL Axioplan2i, Carl Zeiss, Oberkochen, Germany). To detect the resorufin product, a laser tuned to 534 nm was used for excitation, and emission was collected through a 560-nm filter. The master gain, digital gain and digital offset of the fluorescence imaging channel were fixed manually at a proper level during each experiment.

### Droplet disruption by hexane extraction

The droplets in emulsion were recovered by centrifuging at 20,600 × *g* for 1 min at 25°C. The oil phase was removed, and 400 μL of a PBS/T buffer (pH 7.4; PBS containing 0.05% Tween20) was added to it. After a short vortex and spin down, 1 mL of hexane was added to the mixture. Then, the residual oil and surfactant were extracted to the hexane phase by vortexing for 15 s. The hexane phase was separated and removed by centrifuging at 20,600 × *g* for 2 min at 25°C. This hexane extraction step was repeated 3–5 times until the middle layer could not be observed between the other two phases after centrifugation. Next, the recovered microbeads were washed once with 1 mL of PBS buffer, and the activity of the immobilized HRP was detected using the tyramide-based fluorogenic assay.

### Generation of the inactive HRP mutant by using site-directed mutagenesis

The inactive mutant of HRP (MUT) containing two mutations, viz. H170A and T171S, was constructed as a negative control by using the QuickChange site-directed mutagenesis kit (Agilent Technologies, Santa Clara, CA, USA) and two complimentary oligonucleotides (HRP-H170A-S: 5′-ctggt ggcgt tgtct ggtgg tgcaa gcttc gggaa aaacc agtg-3′ and HRP-H170A-AS: 5′-cactg gtttt tcccg aagct tgcac cacca gacaa cgcca ccag-3′). The correct sequence of the plasmid construct was confirmed by DNA sequencing.

### Coupling the DNA template and the scCro-binding hairpin with the microbeads

The HRP/scCro fusions-encoding DNA templates T2 were amplified from the DNA templates T1 (10 ng) and modified with an amino group at the 5′ end of the anti-sense strand in a 20-μL PCR mixture with 0.05 U/μL *Pyrobest* DNA polymerase (Takara) and 0.5 μM of each primer (P2: 5′-ctgcc ccggg ttcct cattc t-3′ and P1-NH_2_: 5′ amino modified oligonucleotide, 5′-ccact acgcc tccgc tttcc tctct atg-3′) using the following temperature sequence: preheating at 94°C for 1 min; followed by 20 cycles of 94°C for 15 s and 70°C for 2 min, with an additional extension at 72°C for 7 min. The PCR products were purified with the QIAquick PCR Purification Kit (Qiagen).

The solution containing the CA microbeads (2.5 μL, 5 × 10^6^ beads) (Dynabeads M-270 carboxylic acid, Life Technologies) was washed twice with 0.01 N NaOH by mixing, using a rotator at room temperature. The microbeads were then washed three times with sterilized water and twice with 2-morpholinoethanesulfonic acid (MES) buffer (200 mM, pH 5.0) in the same manner and the supernatant was discarded. Then, the microbeads were suspended in a 25-μL solution containing 10 μM scCro-binding O_R_ consensus (ORC) hairpin [32] (ORC-NH_2_, 5′ amino modified oligonucleotide, 5′-gatcc tatca ccgcg ggtga tagta cgttt tttcg tacta tcacc cgcgg tgata ggatc-3′) and 150 nM DNA template T2 in MES buffer (100 mM, pH 5.0). The solution was mixed using a rotator at room temperature for 30 min. After mixing, 1.5 mg of 1-ethyl-3-(3-dimethylaminopropyl) carbodiimide hydrochloride (EDC) was added to the microbead solution. The solution was then mixed using a rotator for another 2 h at room temperature. Then, the microbeads were washed thrice with 200 μL of TE buffer containing 0.05% Tween 20 for 10 min, once with TE buffer, and finally suspended in 20 μL of TE buffer for subsequent storage at 4°C.

### Tyramide-based fluorogenic assay

The biotin-labeled tyramide (biotin-tyramide) was prepared according to the published protocol [33]. The activity of HRP on every microbead was measured using an assay mixture containing 50 mM Tris-HCl (pH 7.6), 150 mM NaCl, 200 μM H_2_O_2_, and 8.5 μM biotin-tyramide. The HRP-immobilized microbeads (up to 10^6^ beads) were incubated with 200 μL of the assay mixture 37°C for 10 min, and then washed thrice with 150 μL PBS.

Next, the HRP-microbeads were incubated in 50 μL of Can Get Signal Immunoreaction Enhancer Solution 1 (Toyobo, Osaka, Japan) with 2 μg/mL anti-His antibody (Alexa 488 conjugated, Qiagen) and a 1:100 dilution of streptavidin-Cy5 (Life Technologies) at room temperature for 30 min. The HRP-microbeads were then collected and suspended in 500 μL PBS. The fluorescence intensity on each microbead was analyzed using a flow cytometer (JSAN, Bay Bioscience, Kobe, Japan).

### FACS analysis and sorting

The microbeads were analyzed or sorted by using a flow cytometer. To detect the Cy5 fluorescent dye, a laser tuned to 638 nm was used for excitation, and the emission was detected through a 668–722 nm filter. To detect the Alexa 488 fluorescent dye, a laser tuned to 488 nm was used for excitation, and the emission was detected through a 512–558 nm filter. The microbeads were analyzed at rates of 1000 events per second, and sorted at rates fewer than 500 events per second. Data were analyzed using FlowJo (TreeStar, Ashland, OR, USA).

### Amplification of the microbead-immobilized DNA (Bead PCR)

The immobilized DNA on the microbeads (approx. one thousand) was amplified in a 20-μL PCR mixture with 0.05 U/μL *LA Taq* HS DNA polymerase (Takara) and 0.5 μM of each primer (P2 and P1: 5′-ccact acgcc tccgc tttcc tctct atg-3′) using the following temperature sequence: preheating at 94°C for 3 min; 30 cycles at 94°C for 15 s, 68°C for 2.5 min, and an additional extension at 72°C for 7 min.

## Results and Discussion

### Synthesis of HRP by using the *E*. *coli*-based CFPS system

**Fig 2:**
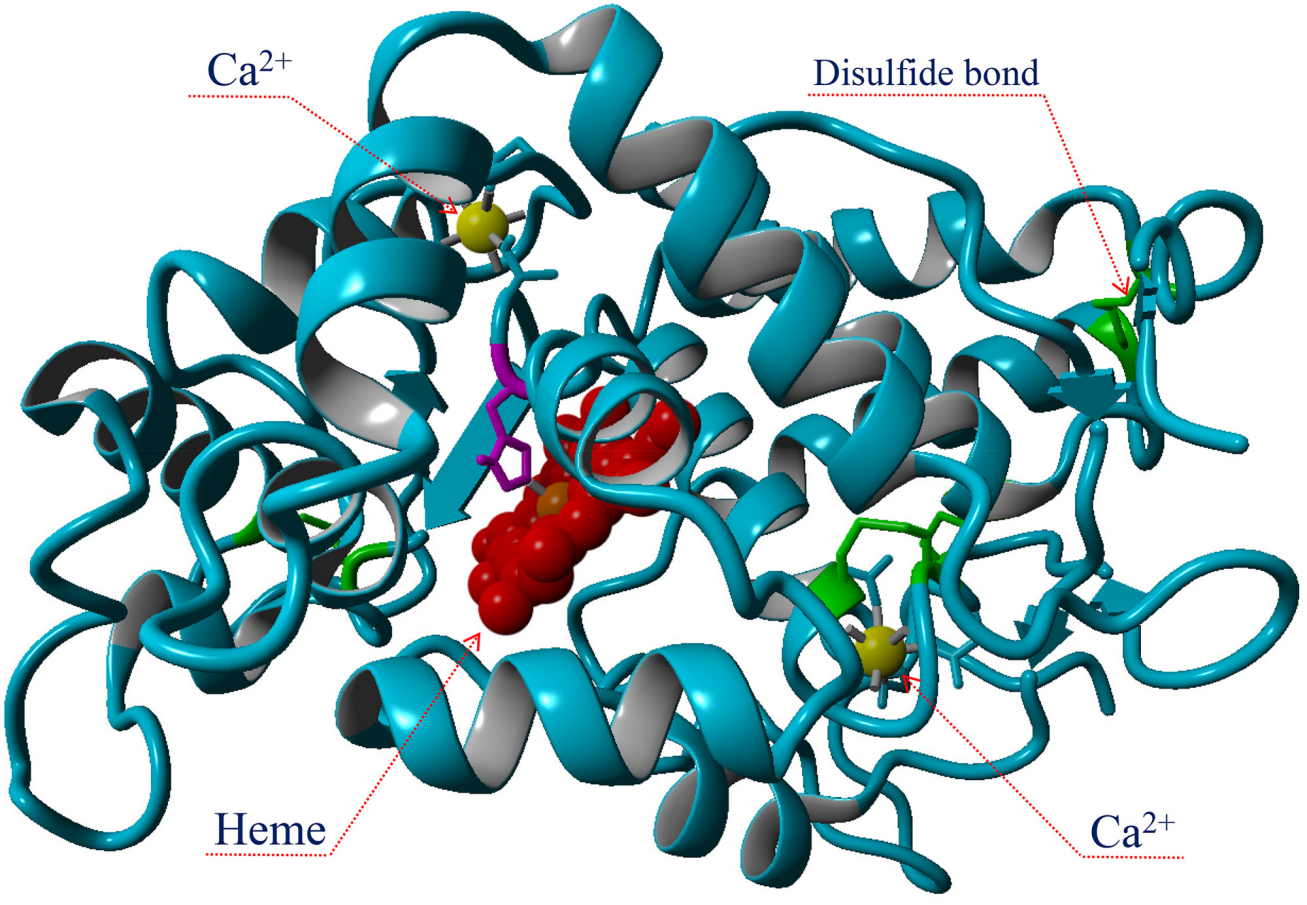
The three-dimensional structure of the horseradish (*Armoracia rusticana*) peroxidase isoenzyme C1a (Protein Data Bank: 1ATJ).

Because HRP is a heme-containing protein and because the heme group is involved in electron transfer during catalysis, varying amounts of hemin were added into the cell-free synthesis reaction. HRP also harbors two calcium-binding sites; one distal and one proximal to the heme group (Fig 2). The removal of calcium from HRP adversely affects not only the enzymatic activity and the thermal stability, but also the structure of the heme pocket [34]. Therefore, the calcium acetate concentration in the cell-free reaction was also examined. An improvement in the productivity of active HRP was obtained when disulfide bond isomerase DsbC was co-expressed with HRP in *E*. *coli* [35]. Therefore, DsbC was utilized in the CFPS reaction.

**Fig 3:**
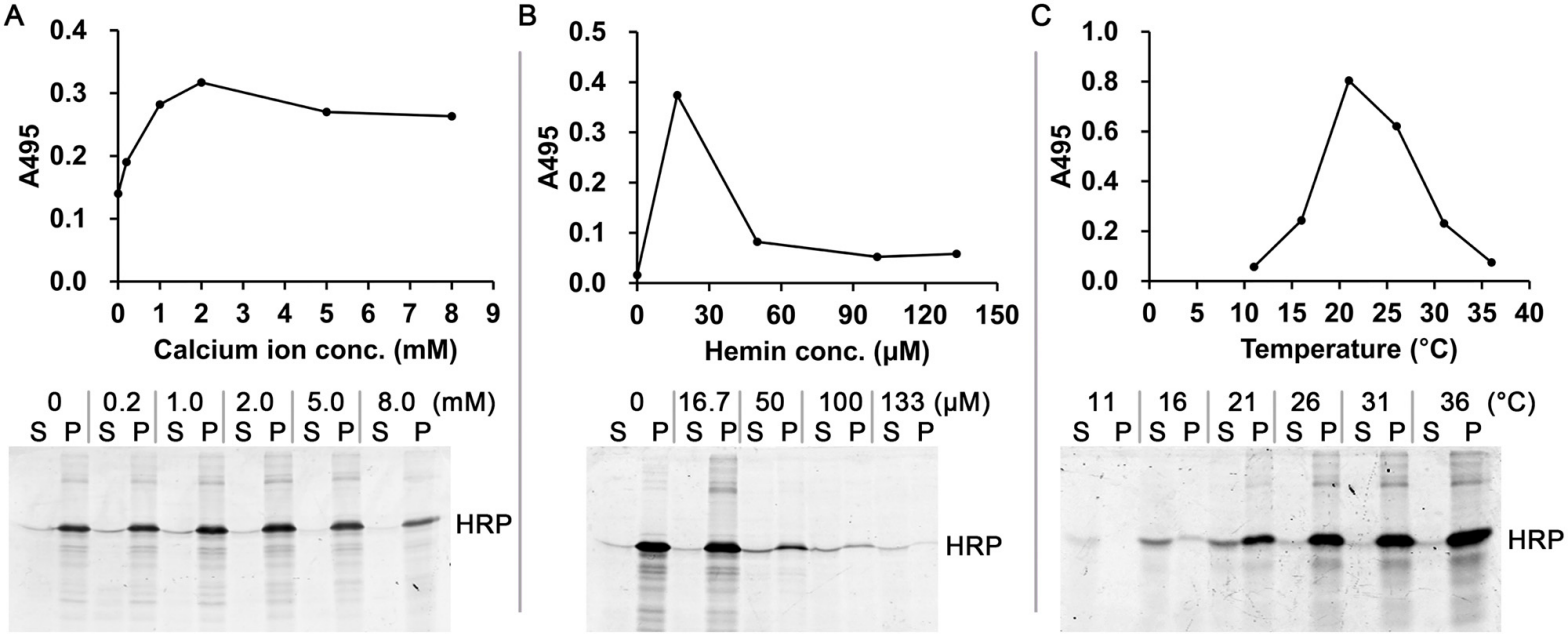
Optimization of the reaction conditions for the CFPS of HRP. A: calcium ions concentration. B: hemin concentration. C: temperature. Top: The activity of the HRP in the CFPS mixture measured by the OPD assay. The coefficient of variation for each data point was <0.05 (calculated from triplicate measurements). Bottom: Soluble (S) and insoluble (P) fractions of the HRP were analyzed by SDS-PAGE with subsequent fluorography.

A survey of the various calcium concentrations revealed that 2 mM calcium acetate provided the highest activity of the synthesized HRP (Fig 3A). Although the activity of HRP synthesized in 2 mM calcium acetate was higher than that of HRP synthesized in 1 mM calcium acetate, the higher calcium concentration inhibited the HRP synthesis in the cell-free system. The effect of different hemin concentrations on HRP synthesis was also tested (Fig 3B). Accordingly, 16.7 μM hemin was considered to be the optimal condition for HRP synthesis. This concentration provided the highest activity, but not the highest solubility. Higher hemin concentration did not increase the synthesis of the active HRP, rather decreased the total amount of the synthesized enzyme. A similar effect had also been observed in our previous study on the CFPS of MnP [23]. This might be related to a change in the redox state of the CFPS reaction by the oxidation potential of hemin. The reaction temperature was another important factor that affected the activity and solubility of the synthesized HRP (Fig 3C). HRP synthesized at 21°C showed both higher activity and solubility than that synthesized at 26°C or 36°C, which are the more common temperatures used during CFPS. This could be caused by the lower rate of translation at low temperatures, which would allow more time for correct protein folding.

HRP was synthesized under the optimized conditions mentioned above, and purified by using the HA-tag purification kit. The specific activity to OPD was approx. 76% of that of the commercially available native glycosylated enzyme. Because the purity of HRP after purification is only around 30% (analyzed by ImageJ), the result is only an estimate. In addition, the lower specific activity might be related to the addition of 3×HA-tag and 6×His-tag or the aglycosylation of the synthesized HRP.

### Selection of an optimal oil and surfactant combination for the CFPS of HRP in emulsion

**Fig 4:**
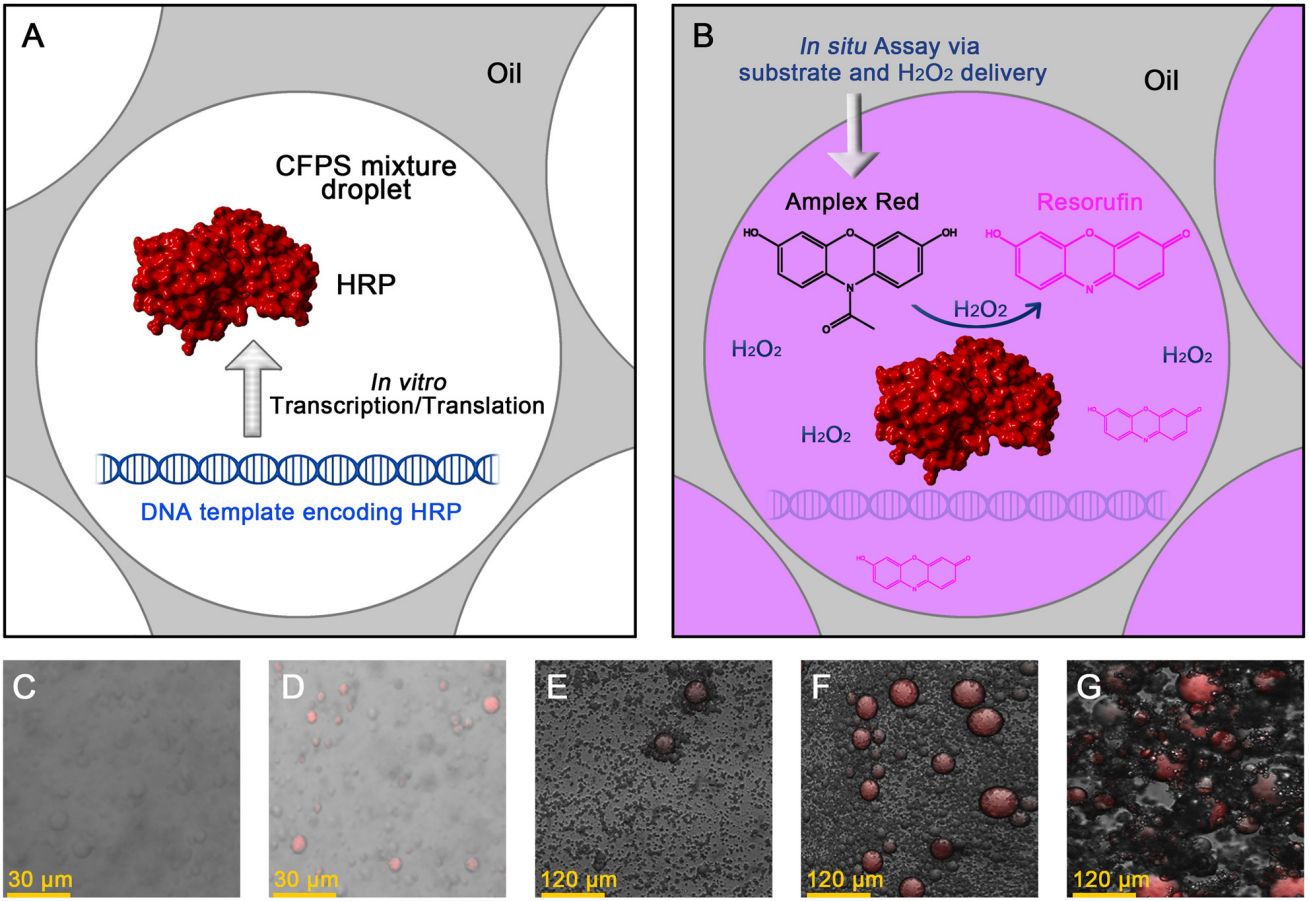
Selection of the appropriate oil/surfactant components during CFPS in emulsion. A-B: Scheme of the *in situ* activity assay for HRP. A: The HRP is synthesized in the emulsion droplet containing the CFPS mixture. B: The substrate, Amplex Red, and H_2_O_2_ are delivered into the droplet. Together with H_2_O_2_, the active HRP converts the non-fluorescent Amplex Red to fluorescent resorufin. C-G: Effects of oil and surfactant combination on the emulsion CFPS. The droplet images were taken using a confocal laser scanning microscope. The data shown in panel C and D were measured during one experiment, while the data shown in panel E, F and G were measured during another separate experiment. C: mineral oil, Span80 (4.5% w/v) and Triton X100 (0.5% w/v); D: hexadecane, Span80 (4.5% w/v) and Triton X100 (0.5% w/v); E: hexadecane and Span80 (5% w/v); F: hexadecane and Span80 (3% w/v); G: hexadecane and SunSoft No. 818SK (3% w/v).

CFPS of the protein of interest in emulsion is one of the core steps in the bead display-based uHTS system (Fig 1). It has been reported that the successful synthesis of a protein by CFPS in emulsion is partially determined by the choice of an optimal oil phase and surfactant [36]. An *in situ* assay (Fig 4A and 4B) was established to detect the expression level of the active HRP in different kinds of emulsions, by using the substrate delivery method described in the Materials and Methods. Fig 4C and 4D show the comparison of mineral oil and hexadecane in the CFPS of HRP in emulsion. HRP synthesized in a hexadecane-based emulsion showed activity in this assay, while no activity could be detected for HRP synthesized in a mineral oil-based emulsion. This result is in accordance with the study on the CFPS of GFP in emulsion [36]. An unknown component of the mineral oil could make CFPS in emulsion unfavorable, especially for the proteins that are not easy to synthesize or fold (e.g., HRP). By contrast, the hexadecane, as a pure alkane, seems more suitable for the CFPS in emulsion than mineral oil, as long as the synthesis reaction temperature is >18°C (melting point of hexadecane).

Another experiment was done to compare the effects of the surfactants Span 80 and SunSoft No. 818SK on HRP synthesis in the hexadecane-based emulsion (Fig 4E, 4F and 4G). The HRP synthesized in emulsion with 3% SunSoft No. 818SK showed the highest activity among these three conditions. The interaction between the proteins and the interface of the droplet, including adsorption and denaturing, has been identified as a major reason why the oil and surfactant can strongly influence the synthesis of proteins in emulsions [36]. The results here support the fact that the oils and surfactants need to be carefully selected for a successful emulsion-based CFPS. The successful *in situ* detection of the HRP synthesized in the droplets could serve as an example for the rapid evaluation of oils/surfactants in the emulsion-based CFPS of other enzymes, without the inclusion of additional influential factors.

### Expression of HRP and scCro fusion proteins

**Fig 5:**
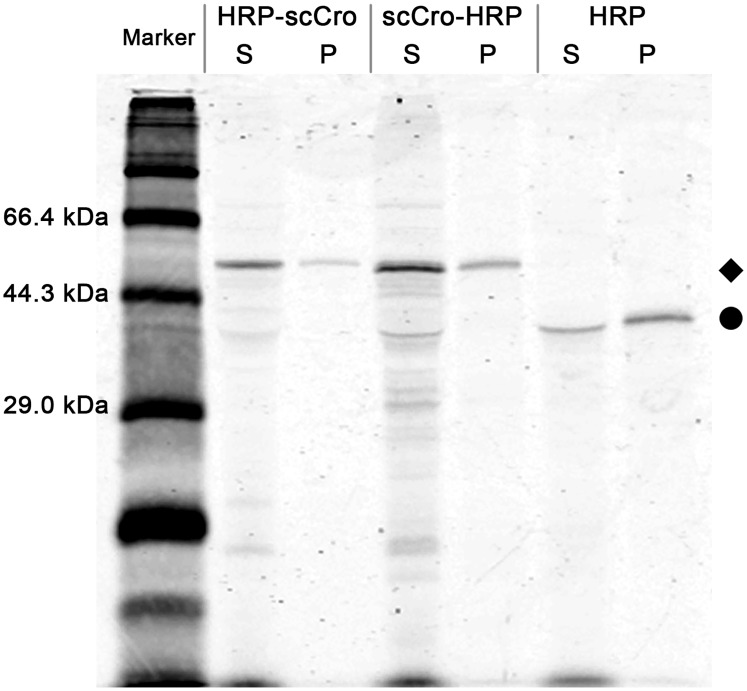
Synthesis of different HRP/scCro constructs in the CFPS system. Soluble (S) and insoluble (P) fractions of HRP/scCro fusion proteins and HRP were analyzed using SDS-PAGE, followed by fluorography. The diamond and circle symbols indicate the positions of HRP/scCro fusions (54 kDa) and HRP (36 kDa) protein bands, respectively.

The scCro binds ORC DNA as a 1:1 complex with a high affinity (*K*_D_ ~ 14 pM) [32]. To achieve stable immobilization of the HRP onto the microbeads, the DNA-binding tag, the scCro-tag, was genetically fused to the N-terminus or C-terminus of HRP with a flexible linker (GSGGGS). A T7-tag was also added on the N-terminal of fusion proteins to improve the protein synthesis level. Then, the HRP/scCro fusion proteins were synthesized under the same conditions as used for the CFPS of HRP. Interestingly, Fig 5 shows that, together with the T7-tag, the scCro-tag at both positions increased the solubility of HRP. The scCro protein can be expressed in a soluble form in *E*. *coli* [27]. This soluble fused partner may elongate the lifetime of the unfolded or misfolded HRP in the cell-free mixture before it accumulates in inclusion bodies. This could secure more time for the disulfide bond isomerase-mediated refolding, and could eventually increase the soluble ratio of the synthesized HRP.

The activities of the fusion proteins were measured with the OPD assay, and were normalized using the relative enzyme amount calculated in ImageJ. The specific activity of HRP-scCro is 11.5% higher than that of scCro-HRP. However, the specific activities of HRP-scCro and scCro-HRP fusions were 13.6% and 12.2% of that of HRP, respectively. It has been reported that a fusion of HRP and a cellulose-binding domain, which had been refolded from inclusion bodies, showed a significantly decreased specific activity (12% of that of the refolded HRP) [37]. Therefore, the scCro-tag might affect the activity of the HRP in a similar way even in the presence of flexible linker.

### Tyramide-based fluorogenic assay for HRP displayed on microbeads

**Fig 6:**
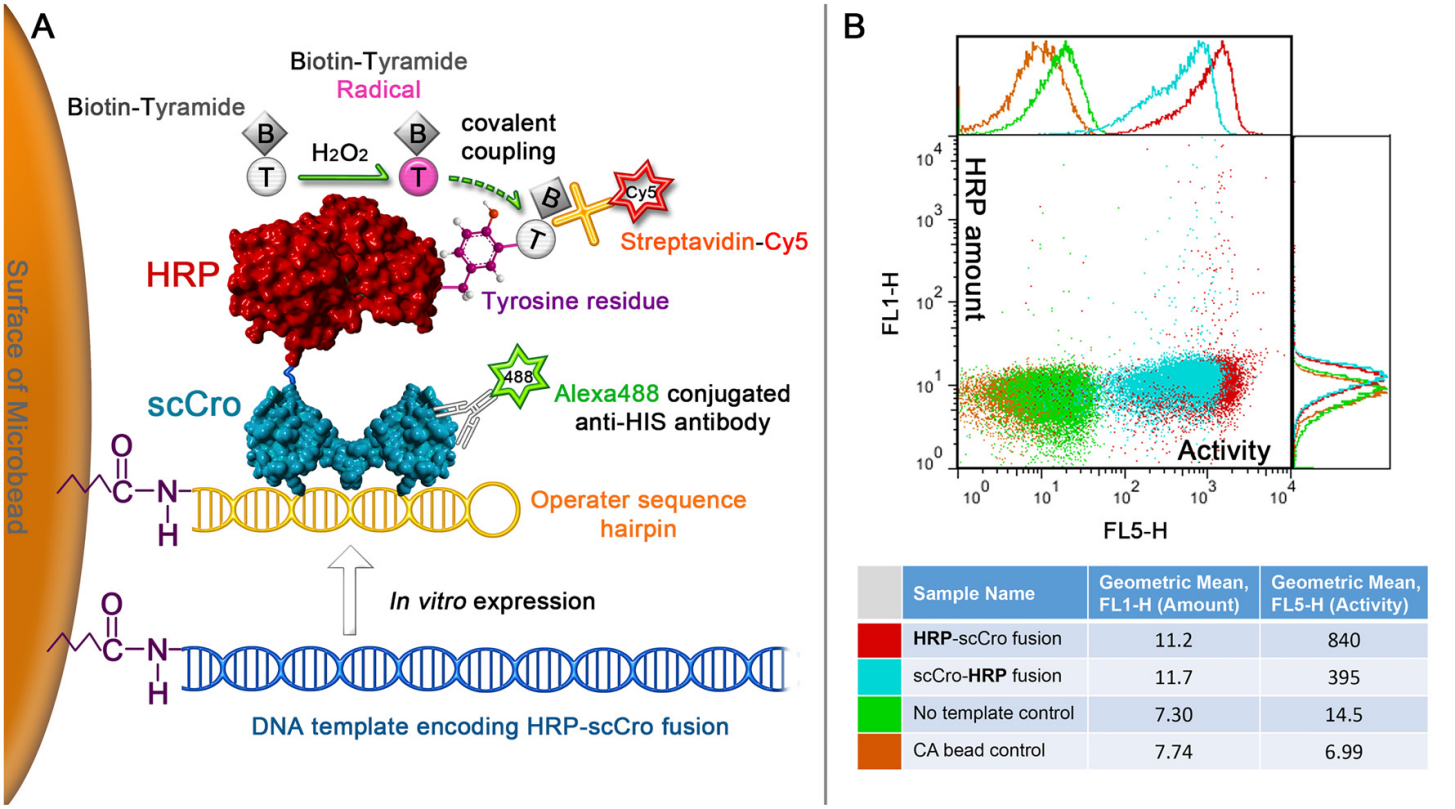
A tyramide-based fluorogenic assay for the HRP displayed on microbeads. (A) Scheme corresponding to the fluorogenic assay. In the presence of H_2_O_2_, HRP catalyzes the conversion of the biotin-labeled tyramide to the short-lived tyramide radical, which can subsequently form a covalent bond with a nearby tyrosine or tryptophan residue on the HRP surface. Streptavidin-Cy5 is then used to indicate the amount of the coupled tyramide. The amount of the immobilized His-tagged HRP-scCro fusion is detected using an Alexa 488-conjugated anti-His tag antibody. (B) Dot-plot results of the flow cytometric analysis.

The role of a fluorogenic assay is to generate a fluorescent signal (related to the activity of the displayed enzyme on the microbead) that could be used in a FACS-based uHTS. The enzyme and its fluorescent signal also need to be present on the same microbead. In order to satisfy both of these requirements, the combination of the biotin-labeled tyramide and the Cy5-labeled streptavidin was chosen as an indicator of HRP activity. In the presence of H_2_O_2_, HRP can catalyze the conversion of tyramide to the short-lived tyramide radical, which can form a covalent bond with the nearby electron-rich moieties (e.g., tyrosine and tryptophan) on the HRP surface [38]. Because the lifetime of the activated tyramide is very short, the cross-interaction between different microbeads is negligible even without compartmentalization. Finally, the amount of 6×His-tagged HRP on the microbead was quantified using an Alexa 488-conjugated anti-His tag antibody to perform a screening for the specific enzyme activity of each microbead (Fig 6A).

The microbeads displaying HRP were mixed with the tyramide assay mixture and incubated for 10 min. Then, the microbeads were analyzed by flow cytometry (Fig 6B). The blue and red dots show the fluorescent signal of the microbeads displaying scCro-HRP and HRP-scCro, respectively, while the green dots represent the signal of the microbeads without HRP. In this assay, the HRP with the C-terminal scCro-tag (HRP-scCro) shows a higher specific activity than the HRP with the N-terminal scCro-tag. This is consistent with the result from the OPD assay described in the last section. Therefore, the HRP-scCro construct was used in the following experiments. The successful simultaneous detection of activity and amount of displayed HRP makes the specific activity-based screening possible by using FACS.

### Expression and display of HRP-scCro fusion in emulsions

**Fig 7:**
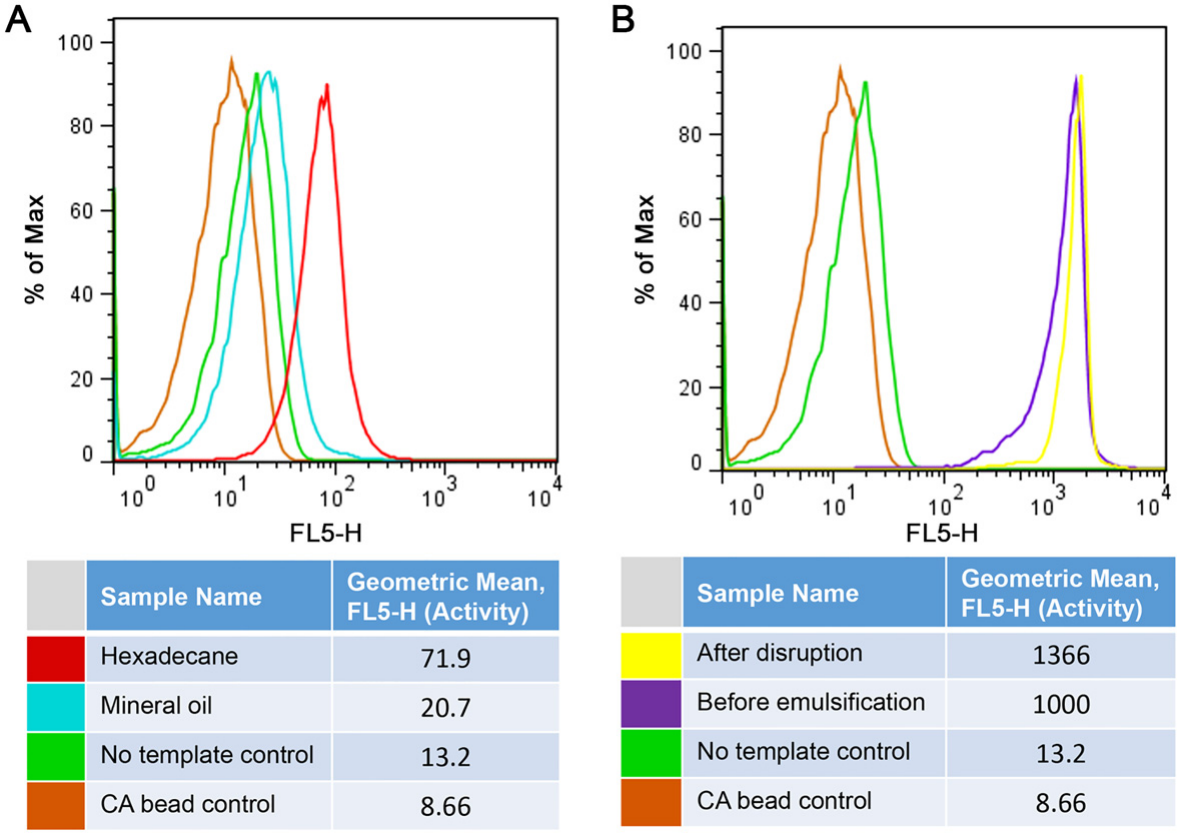
Effects of oil usage and droplet disruption on the HRP-scCro bead display in emulsion CFPS. A: effect of oil usage in emulsion CFPS; B: effect of the disruption step using hexane. Before emulsification: the HRP immobilized microbeads were subjected to the fluorogenic assay without emulsification and droplet disruption steps. After disruption: the HRP immobilized microbeads were subjected to the fluorogenic assay after emulsification and droplet disruption steps.

The optimized combination of the oil phase and the surfactant was further tested and verified during the synthesis of the HRP-scCro fusion by using the tyramide-based fluorogenic assay. The CFPS reaction mixture containing an excessive amount of the HRP-scCro template as well as the microbeads (with the scCro-binding hairpin) was emulsified in hexadecane or mineral oil with 3% SunSoft No. 818SK. At the end of the synthesis, the microbeads were recovered and analyzed using the tyramide-based fluorogenic assay. Fig 7A shows that the fluorescent intensity of the microbeads with the HRP synthesized in the hexadecane-based emulsion is approximately 3.5-fold higher than that synthesized in a mineral oil-based emulsion.

To understand the effect of emulsification and droplet disruption on the displayed HRP, the HRP-scCro was first immobilized on the microbeads in the bulk CFPS reaction. The CFPS mixture was then emulsified by vortexing. The emulsion was incubated at room temperature for 10 min followed by droplet disruption, as described in Materials and Method. Fig 7B shows the fluorescent histogram of the microbeads analyzed by flow cytometry after the tyramide-based fluorogenic assay. This result indicates that the emulsification and droplet destruction does not affect the activity of displayed HRP.

The purpose of performing CFPS (or PCR) in emulsion droplets is to guarantee the linkage of the genotype and phenotype on the same microbead. However, the conditions for single molecule emulsion PCR or single bead emulsion CFPS, such as number of DNA templates or microbeads, are chosen according to the statistical estimation of the Poisson distribution [24]. This method cannot completely exclude the contamination between different HRP mutants, especially when the emulsion is generated by vortexing, which leads to non-uniform droplet sizes. However, this possibility can be reduced by using a flow-focusing device to generate more uniform emulsion droplets.

### Screening of a model microbead library

**Fig 8:**
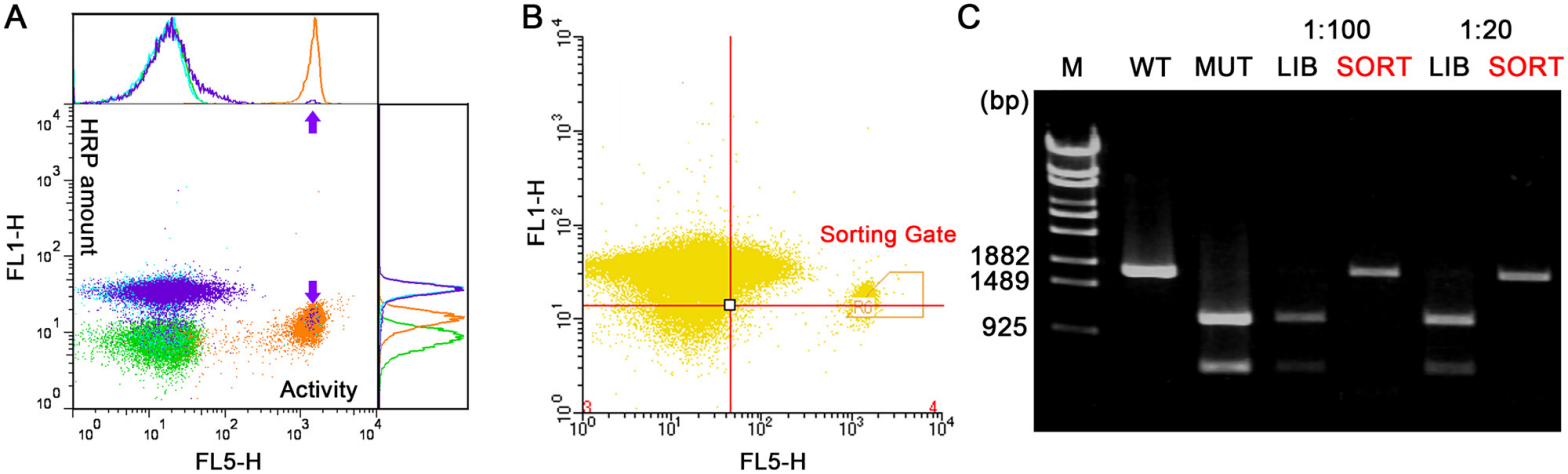
Enrichment of the wild type *hrp* gene from a model library using FACS. A: Flow cytometric analysis of the positive control (orange), negative control (blue), and the 1:100 model library (purple) after the fluorogenic assay. Green dots indicate the no template control. The peak and dots representing the wild type HRP events in the model library are indicated by purple arrows. B: Gate selection during the sorting of the model library. The yellow trapezoid indicates the gating region for collecting the microbeads with active HRP. The sorting continued until around one thousand microbeads were obtained. C: Electrophoretic analysis of the enriched DNA after the sorting. M: λ-*Eco*T14 I digest DNA marker; WT: wild type; MUT: inactive mutant; LIB: model library; SORT: sorted microbeads.

His170 in HRP is an important residue for binding of the heme prosthetic group, and the activity of the H170A mutant towards ABTS is only 0.004% [39]. Therefore, an inactive mutant containing two point mutations, H170A and T171S (for introducing a *Hin*dIII site), was constructed as a negative control. Two kinds of microbeads chemically coupled with DNA of wild type HRP (positive control) or its inactive mutant (negative control) were prepared separately. The *in vitro* synthesized HRP or its inactive mutant in bulk was then immobilized on the corresponding microbeads. The positive and negative control microbeads were mixed in a 1:100 ratio. Then, two kinds of control microbeads and the model library were subjected to the fluorogenic assay, and analyzed (Fig 8A) or screened using a cell sorter (Fig 8B). Approximately 1000 microbeads (0.5% of total events) were sorted in gate R6 of Fig 8B, and the DNA on these microbeads was amplified by bead PCR. The PCR products were treated with the *Hin*dIII restriction enzyme, and analyzed via electrophoresis.

In Fig 8A, the orange and blue dots represent the positive and negative controls, respectively. The model library microbeads (purple dots) contain two separate populations, which indicates that the cross-interaction between the two kinds of microbeads is minimal in the bulk assay reaction. The electrophoresis result (Fig 8C) indicates a highly effective enrichment of the wild type *hrp* gene from the model library. The amplified DNA was then cloned into the pGEM-T vector and transformed into the *E*. *coli* strain DH5α. All ten ‘valid’ colonies (containing the plasmid carrying the wild type or mutated hrp gene) tested by colony PCR contained the wild type *hrp* gene (S3 Fig), which proved that the wild type gene dominated in the enriched DNA from one round of screening using this fluorogenic assay.

In conclusion, by optimizing the reaction conditions and the oil/surfactant components, the active HRP with the scCro-tag was successfully synthesized both in bulk and in emulsion. We also demonstrated the feasibility of the tyramide-based fluorogenic assay by screening of a 1:100 model microbead library, resulting in a highly effective enrichment of the active *hrp* gene. Before using this uHTS platform in the screening of a random mutation library, the emulsion PCR (Fig 1B) for HRP must be optimized to improve the efficiency of the amplification of large DNA fragments like the HRP/scCro encoding template DNA on microbeads by a survey of the most suitable DNA polymerase [40]. In addition, to avoid a decrease of signal caused by a reduced number of accessible surface Tyr/Trp residues in HRP mutants, a poly-Tyr peptide could be added to the scCro-tag to provide additional binding sites for the tyramide radical. These additional binding sites could also improve the resolution of the specific activity measurement of this platform, which can make the comparison of different HRP mutant easier during the screening of a random mutation library. In general, the selection of an improved mutant might be difficult in the screening of a random mutation library, because the evolution of each property of the enzyme is not always synchronous. However, as a specific activity-based uHTS platform for HRP, it should be effective in the first screening of a large mutant library.

The compatibility of the tyramide-based fluorogenic assay to another peroxidase, viz. MnP from *Phanerochaete chrysosporium*, has been confirmed in our laboratory. It indicates that the fluorogenic assay and uHTS platform described here can be applied to other peroxidases easily, with minimal modifications. In addition to activity-based screening, this platform is also suitable for improving the industrially important properties of target enzymes (e.g., thermostability, soluble expression level, and tolerance for conditions) in an ultra-high-throughput manner.

## Supporting Information

S1 File Amino acid sequence of the mature HRP. (DOC)

S2 File The complete sequence of the synthetic *hrp* gene optimized for the CFPS. (DOC)

S3 File The sequence from T7 promoter to T7 terminator of the plasmid pRSET-HRP-scCro. (DOC)

S1 Fig The schematic diagram of the construction of the synthetic *hrp* gene optimized for the CFPS. (TIF)

S2 Fig The schematic diagram of the construction from T7 promoter to T7 terminator of plasmid pRSET-HRP-scCro. (TIF)

S3 Fig Electrophoresis of the colony PCR products. M: λ-*Eco*T14 I digest DNA marker; 1: gene of the wild type HRP, positive clone; 2: gene of the HRP inactive mutant, negative clone; 3–15: thirteen randomly chosen colonies; red arrow indicates the correct size of the positive clone. The DNA bands in lanes 3, 9, and 11 indicate non-specific amplification from the sorted microbeads (non-valid clone).
(TIF)
