# Supplementary material for: Ultra-High-Throughput Screening of an In Vitro-Synthesized Horseradish Peroxidase Displayed on Microbeads Using Cell Sorter
Source: PLoS One. 2015 May 20;10(5):e0127479. doi: 10.1371/journal.pone.0127479 (PMC4439038; doi:10.1371/journal.pone.0127479)
Supplement: S1 File — (DOC) [file pone.0127479.s001.doc]

>HRP 308 Horseradish peroxidase [*Armoracia rusticana*]

QLTPTFYDNSCPNVSNIVRDTIVNELRSDPRIAASILRLHFHDCFVNGCDASILLDNTTSFRTEKDAFGNANSARGFPVIDRMKAAVESACPRTVSCADLLTIAAQQSVTLAGGPSWRVPLGRRDSLQAFLDLANANLPAPFFTLPQLKDSFRNVGLNRSSDLVALSGGHTFGKNQCRFIMDRLYNFSNTGLPDPTLNTTYLQTLRGLCPLNGNLSALVDFDLRTPTIFDNKYYVNLEEQKGLIQSDQELFSSPNATDTIPLVRSFANSTQTFFNAFVEAMDRMGNITPLTGTQGQIRLNCRVVNSNS
