# Supplementary material for: Ultra-High-Throughput Screening of an In Vitro-Synthesized Horseradish Peroxidase Displayed on Microbeads Using Cell Sorter
Source: PLoS One. 2015 May 20;10(5):e0127479. doi: 10.1371/journal.pone.0127479 (PMC4439038; doi:10.1371/journal.pone.0127479)
Supplement: S2 File — (DOC) [file pone.0127479.s002.doc]

atctcgatcccgcgaaattaatacgactcactatagggagaccacaacggtttccctctagaaataattttgtttaactttaagaaggagatatacat

T7 promoter

rbs

ATG

HA-tag

TATCCTTATGATGTGCCTGACTACGCG

G spacer

GGT

HA-tag

TACCCATATGACGTACCAGATTATGCA

GS spacer

GGA TCC

HA-tag

TATCCGTACGATGTTCCGGATTACGCC

8 × G linker

GGTGGAGGCGGTGGAGGTGGAGGT

CAACTCACCCCAACTTTCTACGACAATTCATGCCCGAACGTTAGCAACATTGTCCGCGACACCATCGTAAACGAACTGCGTTCTGATCCGCGTATTGCTGCGTCCATCCTGCGCCTGCACTTCCACGATTGTTTCGTTAACGGATGCGACGCGTCTATCCTGCTGGACAACACCACCTCCTTCCGTACCGAAAAGGATGCGTTCGGCAACGCCAACTCCGCGCGCGGTTTCCCAGTTATCGACCGCATGAAAGCCGCTGTAGAATCCGCCTGTCCGCGTACCGTATCTTGCGCAGACCTCCTGACCATCGCGGCCCAGCAGAGCGTTACTCTAGCAGGTGGCCCGTCTTGGCGTGTTCCGCTGGGTCGTCGTGATTCTCTACAGGCGTTCCTGGATCTGGCCAACGCAAATCTGCCAGCTCCGTTCTTCACCCTGCCGCAGCTGAAAGATAGCTTCCGTAACGTTGGCCTGAACCGTTCATCCGATCTGGTGGCGTTGTCTGGTGGTCACACCTTCGGGAAAAACCAGTGCCGTTTCATCATGGACCGCCTGTATAACTTCTCGAACACCGGTCTGCCGGACCCGACCCTGAACACCACCTATTTGCAGACTCTGCGTGGGCTGTGCCCGCTGAACGGTAACCTGTCCGCGCTGGTTGACTTCGATCTGCGTACTCCGACCATCTTCGATAACAAATACTACGTTAACCTGGAAGAACAGAAGGGCCTGATTCAGTCTGACCAGGAGCTGTTCTCCTCCCCGAACGCGACCGACACCATCCCGCTGGTTCGTAGCTTCGCGAACAGCACGCAGACTTTCTTCAACGCTTTCGTAGAGGCTATGGACCGTATGGGTAACATTACCCCGCTGACCGGTACGCAGGGACAGATCCGCCTGAACTGCCGCGTGGTTAACTCCAACTCC

HRP

6 × His-tag

CACCACCACCACCACCAC

Double stop condon

TAATAA

gatccggctgctaacaaagcccgaaaggaagctgagttggctgctgccaccgctgagcaataactagcataaccccttggggcctctaaacgggtcttgaggggttttttgctgaaaggaggaactatatccgga

T7 terminator
