# Supplementary material for: Ultra-High-Throughput Screening of an In Vitro-Synthesized Horseradish Peroxidase Displayed on Microbeads Using Cell Sorter
Source: PLoS One. 2015 May 20;10(5):e0127479. doi: 10.1371/journal.pone.0127479 (PMC4439038; doi:10.1371/journal.pone.0127479)
Supplement: S3 File — (DOC) [file pone.0127479.s003.doc]

atctcgatcccgcgaaattaatacgactcactatagggagaccacaacggtttccctctagataattttgtttaactttaagaaggagatatacat

T7 promoter

rbs

ATG

T7-tag

ATGGCGTCTATGACCGGTGGTCAGCAGATGGGT

CAACTCACCCCAACTTTCTACGACAATTCATGCCCGAACGTTAGCAACATTGTCCGCGACACCATCGTAAACGAACTGCGTTCTGATCCGCGTATTGCTGCGTCCATCCTGCGCCTGCACTTCCACGATTGTTTCGTTAACGGATGCGACGCGTCTATCCTGCTGGACAACACCACCTCCTTCCGTACCGAAAAGGATGCGTTCGGCAACGCCAACTCCGCGCGCGGTTTCCCAGTTATCGACCGCATGAAAGCCGCTGTAGAATCCGCCTGTCCGCGTACCGTATCTTGCGCAGACCTCCTGACCATCGCGGCCCAGCAGAGCGTTACTCTAGCAGGTGGCCCGTCTTGGCGTGTTCCGCTGGGTCGTCGTGATTCTCTACAGGCGTTCCTGGATCTGGCCAACGCAAATCTGCCAGCTCCGTTCTTCACCCTGCCGCAGCTGAAAGATAGCTTCCGTAACGTTGGCCTGAACCGTTCATCCGATCTGGTGGCGTTGTCTGGTGGT*CACAC*CTTCGGGAAAAACCAGTGCCGTTTCATCATGGACCGCCTGTATAACTTCTCGAACACCGGTCTGCCGGACCCGACCCTGAACACCACCTATTTGCAGACTCTGCGTGGGCTGTGCCCGCTGAACGGTAACCTGTCCGCGCTGGTTGACTTCGATCTGCGTACTCCGACCATCTTCGATAACAAATACTACGTTAACCTGGAAGAACAGAAGGGCCTGATTCAGTCTGACCAGGAGCTGTTCTCCTCCCCGAACGCGACCGACACCATCCCGCTGGTTCGTAGCTTCGCGAACAGCACGCAGACTTTCTTCAACGCTTTCGTAGAGGCTATGGACCGTATGGGTAACATTACCCCGCTGACCGGTACGCAGGGACAGATCCGCCTGAACTGCCGCGTGGTTAACTCCAACTCC

HRP

GSGGGS linker

GGTTCTGGTGGTGGCTCT

HA-tag

TATCCGTACGACGTGCCGGATTACGCG

ATGGAACAACGCATAACCCTGAAAGATTATGCAATGCGCTTTGGGCAAACCAAGACAGCTAAAGATCTCGGCGTATATCAAAGCGCGATCAACAAGGCCATTCATGCAGGCCGAAAGATTTTTTTAACTATAAACGCTGATGGAAGCGTTTATGCGGAAGAGGTAAAGCCCTTCCCGAGTAACAAAAAAACAACAGCAGCCGGTACCGGTGGCTCTGGCGGCATGGAGCAGCGAATTACTCTTAAGGATTACGCTATGCGATTCGGCCAGACAAAAACTGCAAAGGACCTTGGAGTTTACCAGAGCGCCATTAATAAAGCGATACACGCTGGTCGTAAGATCTTTCTTACAATAAATGCCGACGGTTCTGTTTACGCTGAGGAAGTTAAACCTTTTCCTAGTAATAAGAAAACTACGGCT

scCro-tag

FLAG-tag

GACTACAAAGATGACGACGATAAA

G-Y-S linker

GGT TAC TCT

6 × His-tag

CACCACCACCACCACCAC

TAA

T7 terminator

gatccggctgctaacaaagcccgaaaggaagctgagttggctgctgccaccgctgagcaataactagcataaccccttggggcctctaaacgggtcttgaggggttttttgctgaaaggaggaactatatccgga
