# Supplementary figures and images for: Ultra-High-Throughput Screening of an In Vitro-Synthesized Horseradish Peroxidase Displayed on Microbeads Using Cell Sorter
Source: PLoS One. 2015 May 20;10(5):e0127479. doi: 10.1371/journal.pone.0127479 (PMC4439038; doi:10.1371/journal.pone.0127479)

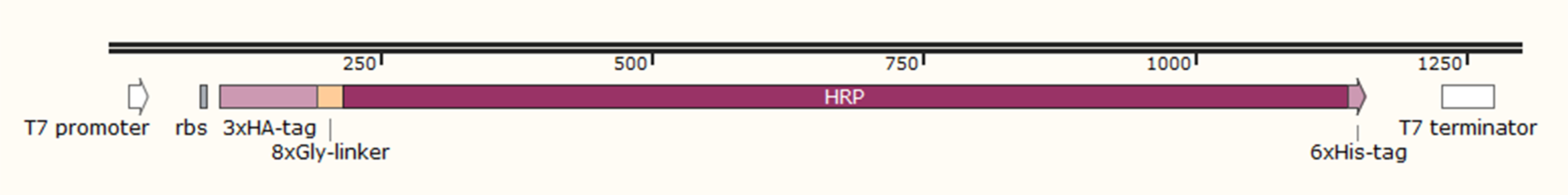

Supplement: S1 Fig — (TIF) [file pone.0127479.s004.tif]

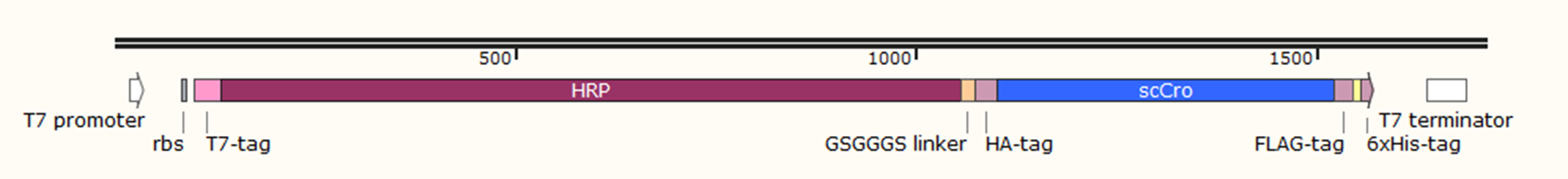

Supplement: S2 Fig — (TIF) [file pone.0127479.s005.tif]

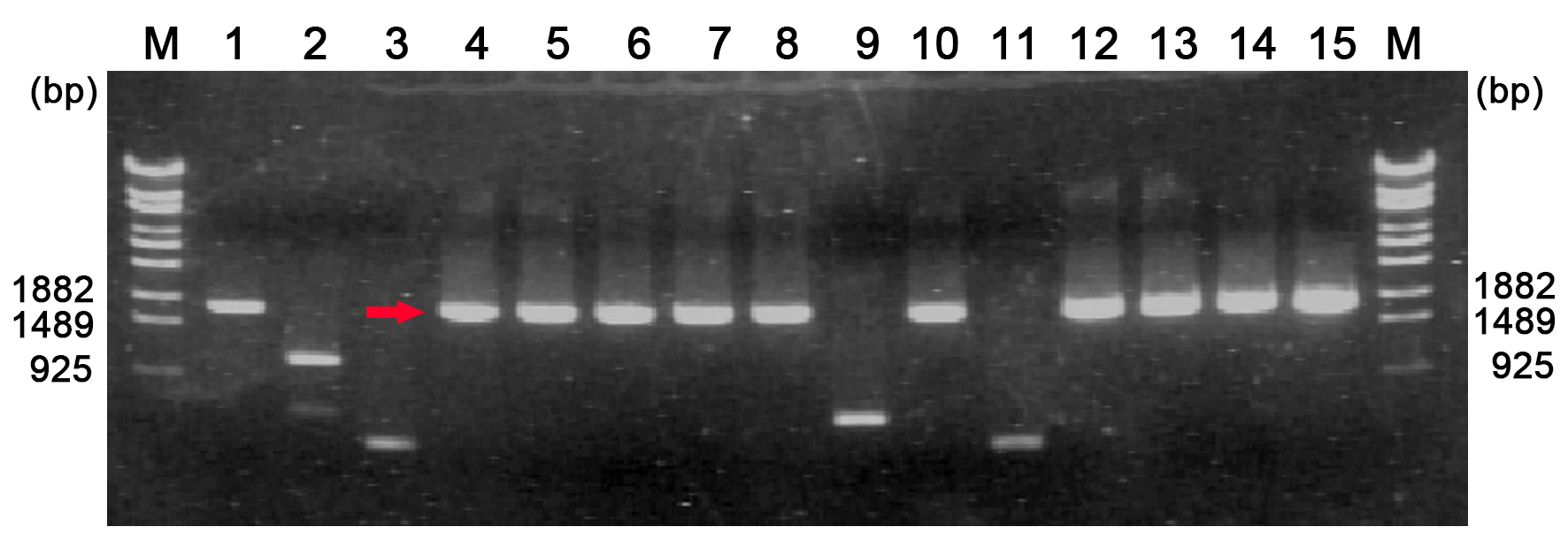

Supplement: S3 Fig — M: λ-EcoT14 I digest DNA marker; 1: gene of the wild type HRP, positive clone; 2: gene of the HRP inactive mutant, negative clone; 3–15: thirteen randomly chosen colonies; red arrow indicates the correct size of the positive clone. The DNA bands in lanes 3, 9, and 11 indicate non-specific amplification from the sorted microbeads (non-valid clone). (TIF) [file pone.0127479.s006.tif]
